# Potential of Bacterial Cellulose in Reconstructive Surgery of Body Integumentary System: Preliminary Studies in Animals

**DOI:** 10.3390/jfb14080397

**Published:** 2023-07-26

**Authors:** Agata Błażyńska-Spychalska, Martyna Kur, Tomasz Brzeski, Wacław Zając, Teresa Pankiewicz, Stanisław Bielecki, Jarosław Woliński, Jerzy Jankau

**Affiliations:** 1 Department of Plastic Surgery, Medical University of Gdańsk, 80-210 Gdańsk, Poland; agata.blazynska@gumed.edu.pl (A.B.-S.); martyna.kur@gumed.edu.pl (M.K.); 2 Independent Researcher, 81-303 Gdynia, Poland; brzeskigdynia@wp.pl; 3 Independent Researcher, 75-671 Koszalin, Poland; wetkicaj@interia.pl; 4 Institute of Molecular and Industrial Biotechnology, University of Technology of Łódź, 90-924 Łódź, Poland; teresa.pankiewicz@p.lodz.pl (T.P.); stanislaw.bielecki@p.lodz.pl (S.B.); 5 The Kielanowski Institute of Animal Physiology and Nutrition, Polish Science Academy, 05-110 Jabłonna, Poland; j.wolinski@ifzz.pl

**Keywords:** bacterial cellulose, reconstructive surgery, body integumentary system, animal studies

## Abstract

The aim of the study is to present the preliminary results of the in vivo application of *Komagataeibacter xylinum* E25 bacterial cellulose (BC) as a replacement material for produced defects during operations. Three pigs (sus scrofa domestica) had the same defects in the ear cartilage (4 × 4 cm) and in the rectus abdominis muscle (6 × 10 cm) with BC membranes implanted into them. The time of observation of the condition of the animals was 3 months. Implantation sites did not show clinical signs of complications in the form of inflammation or necrosis. Histologically, a normal scar was produced as a result of the material healing into the host’s body. In one case, no residual implant material was found at the site of implantation, and the remodeled scar confirmed healing. No systemic inflammatory reaction was observed in any of the animals. The host organism’s reaction to the bacterial cellulose allows us to believe that it meets the expectations as a material that can be widely used in reconstructive surgery. Nevertheless, this requires further research on a larger group and also using other foreign bodies. The next step would be an experiment on a group consisting of people.

## 1. Introduction

In recent years, there has been significant progress in biotechnology, and its increasing application in medicine can be observed. Biotechnology allows for improving results in many medical fields, including surgery. This is particularly noticeable in the field of reconstructive surgery, where the most important problem is the tissue deficit resulting from the defect usually caused by the surgery itself. Doctors for centuries and biotechnologists since the establishment of this discipline, i.e., from the beginning of the 1970s, have been trying to obtain materials to replace lost human tissues as a result of injuries, diseases, and underdevelopment due to congenital defects. A surgical specialty that deals with the repair of body deformities resulting from the above-mentioned causes is plastic and reconstructive surgery. A frequent problem is the lack or shortage of soft tissues of the body’s integumentary system caused by a congenital defect, trauma, or, more and more often, cancer. That is why it is important to find material that allows for easy reconstruction of natural tissues without local inflammatory reaction, which is common when in contact with most foreign materials. The basic method of reconstruction is harvesting the patient’s own tissues (autologous reconstruction methods), but it is often difficult or impossible—e.g., due to the excessive amount of tissue necessary for reconstruction to be successful and functional, or insufficient tissue quality. Autologous reconstructions are also often associated with a large wound surface resulting from the creation of a donor site from which tissues are taken. This can lead to poorer healing and often longer hospitalization, which is not expected or taken well by the patients. Moreover, the transfer of tissue flaps is usually associated with the risk of local ischemia and sometimes ischemia of the whole flap, which carries the risk of local or systemic infection. The risk of complications due to ischemia and tissue harvesting from the donor site also leads to larger scars, which reduces the final aesthetic satisfaction of the patient which is often expected when patients are treated by a plastic surgeon. Therefore, it is desirable to supplement the cavities with entirely artificial materials (implants) or materials of natural origin (biotechnological products). To fulfill their function, these materials must have several features, such as no or minimal immune reaction, good integration with host tissues, ease of modeling, mechanical strength, easy availability, and low cost [1]. The implants used so far may contain metals, organometallic compounds, polypropylene and polyethylene derivatives, or silicone gel compounds. All of these could trigger a local or systemic immune response. At the same time, the knowledge of biomaterials is expanding, among which one of the most interesting and promising is bacterial cellulose (BC) produced by the *Komagataeibacter xylinum* E25 strain [2]. Bacterial cellulose (BC) is a linear polysaccharide composed of β-d-glucopyranose monomers linked by β-1,4-glycosidic linkages. It is an exopolysaccharide free of endotoxins and composed only of biocellulose fibers. The biochemical reactions of BC biosynthesis by *K. xylinus* are well characterized. This is a precise and specifically regulated advanced process, involving many individual enzymes and complexes of catalytic and regulatory proteins. BC is a flexible membrane with high crystallinity, mechanical strength, a high degree of hydration, and thermal stability. It is characterized by a significant possibility of shaping the surface [3]. BC is biocompatible, hypoallergenic, non-mutagenic, and non-teratogenic, similar to the collagen found in the human body. These properties enable BC to be used in regenerative and reconstructive medicine. In addition, its use avoids several problems. These include problems of finding a donor (in the case of allogeneic transplants), damaging the donor site, and creating a wound and excessive scarring (in the case of autogenic transplants), as well as problems with the rejection of transplants [2]. So far, BC has been used to produce dressings with proven clinical effectiveness in human studies and structures that replace or strengthen anatomical structures such as large blood vessels, heart valves, fasciae, or trachea in animal studies [3,4,5,6]. The efficient production of bacterial cellulose meeting the requirements of biomedicine is the subject of many studies summarized in review articles and patents [3]. However, few of them concern experimental work on animals. Considering the above-mentioned features of BC, the aim of the present study was a clinical and histopathological evaluation of a membrane made of bacterial cellulose used to fill defects in the auricle and rectus muscle in a domestic pig animal model. Muscle and cartilage defects are common problems in reconstructive surgery, both in the face and body. Craniofacial injuries very often involve protruding elements, including the nose and ears, which are associated with damage to the cartilaginous parts that constitute the frame. Defects resulting from chronic wounds or resections for oncological reasons are usually associated with large muscle losses. The closure of such defects requires various muscle flaps to be functional. Therefore, the study of a material that would be suitable as a replacement for these tissues is extremely important.

## 2. Materials and Methods

This was an in vivo animal model study carried out on three specimens. Pigs of the Comboro breed *Great White English* (*Sus scrofa domesticus*) were used. The intervention in the present study was the implantation of BC into previously prepared rectus abdominis muscle of the pig and ear of the pig, as described below. Animals were 6 months ± 2 weeks old and weighed 25 kg ± 2 kg (on the day of commencement of the study). All surgical procedures were performed at the Veterinary Clinic in Gdynia after obtaining the permission of the Local Ethical Committee for Experiments on Animals No. 3 at the Medical University of Gdańsk No. 58/2012 of 3 December 2012. The animals were implanted with previously prepared sterile cellulose membranes with the following properties.

### 2.1. Material

The *Komagateibacter xylinus* E25 strain used in the research came from the culture collection of the Institute of Molecular and Industrial Biotechnology (Lodz University of Technology). The strain belongs to the company BOWIL Biotech Ltd., 84-120 Władysławowo, Poland.

### 2.2. Culture Media

The *K. xylinus* E25 strain was grown under stationary conditions in a pilot plant of the Institute of Molecular and Industrial Biotechnology and on Schramm–Hestrin (SH) medium containing glucose (20 g/L), yeast extract (5.0 g/L), peptone (5.0 g/L), MgSO_4_ × 7 H_2_O (0.5 g/L), anhydrous Na_2_HPO_4_ (2.7 g/L), and citric acid (1.15 g/L), dissolved in distilled water. Prior to autoclaving at 121 °C for 20 min, the pH of the culture medium was 5.7 using 0.1 M acetic acid. The sterile medium was replenished with ethanol (1% *v*/*v*) immediately prior to inoculation with a bacterial preculture (5% *v*/*v*) grown in the same medium. Membranes were grown at 30 °C until the desired thickness of the cellulose shell was obtained. Membrane-purification steps were carried out according to the standard BC purification method and included rinsing with tap water to rinse any remaining medium, rinsing for 24 h in 1% *v*/*v* sodium hydroxide to remove bacterial cells, and immersing in 1% *v*/*v* acetic acid and then in distilled water to neutralize the pH [7]. Purified cellulose samples were packaged and sterilized by irradiation prior to further analytical steps and animal testing.

### 2.3. Surgical Procedures

**Figure 1:**
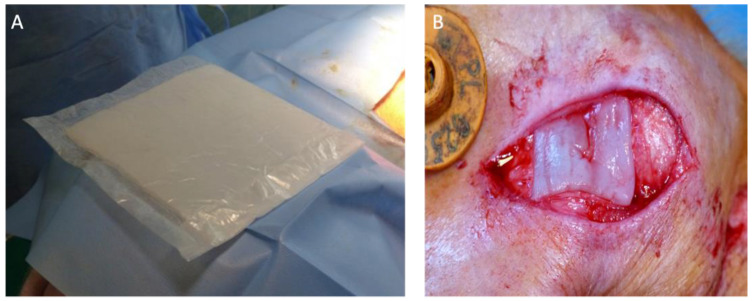
BC matrix in an operating field (**Panel A**). BC matrix implanted into a pig’s ear (**Panel B**).

**Figure 2:**
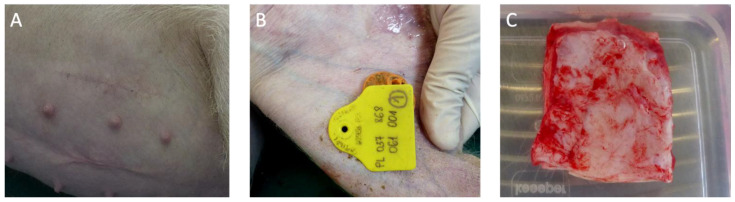
Implantation site on the abdomen after 90 days of observation (**Panel A**). Implantation site on the ear after 90 days of observation (**Panel B**). Material after excision for the histopathological evaluation (**Panel C**).

The living conditions of pigs and the treatments performed on them complied with the applicable standards contained in the Regulation of the Ministry of Agriculture and Rural Development of 25 February 2005, Journal of Laws No. 39, Item 374. The choice of the domestic pig as the test animal was dictated by the animal’s resemblance to humans in terms of anatomy and physiology [8]. Each pig was subjected to surgery under general anesthesia with the following preparations in exact amounts: Scanofol 10 mg/mL, Stresnil 40 mg/mL administered intravenously, and Aerrane/isofluranum inhaled. The operations were performed under aseptic conditions in a properly equipped operating room. BC membranes were implanted in the form of a rectangular material (Figure 1 and Figure 2) within the surgically generated defects: 4 × 4 cm in ear cartilage and 5 × 12 cm in one of the rectus abdominis muscles. BCs were attached to the surrounding tissues with single insoluble 2.0 Prolene (Ethicon, Johnson & Johnson CO) sutures.

### 2.4. Time of Observation

The observation time for each animal was 90 days, during which the animals were kept in individual boxes in order not to damage the inserted recipient sites. The animals were fed according to their food supply age. Moreover, they had constant access to water. After this time, the animals were put down by a veterinarian in accordance with FELASA guidelines [9,10]. The BC material was excised and sent for histopathological examination in a 10% formaldehyde solution. The animals were then disposed of in accordance with FELASA guidelines [9,10].

### 2.5. Clinical Evaluation

Clinical evaluation was performed by 2 experienced plastic surgeons (J.J., A.B.-S.). These surgeons perform reconstructions on a daily basis, both autologous and with the use of synthetic and biological materials available so far in medicine. The evaluation comprised an assessment of signs of inflammation (discoloration, edema, temperature, fever), wound healing (progression of proper scar formation), and the shape and consistency of the graft site.

### 2.6. Histopathological Examination

Tissue material was fixed in 10% formalin for 48 h by standard procedure. Subsequently, the collected sections were dehydrated by passing them through appropriate concentrations of alcohol and xylene, and finally embedded in low-melting paraffin. Sections were cut into 5 µm thick sections that were routinely stained with hematoxylin and eosin (H&E).

## 3. Results

### 3.1. Clinical Evaluation

During the 3-month observation of the animals, no clinical signs of local or generalized inflammatory reaction were found. The animals were in good general condition throughout the observation period, and there was no fever or signs of generalized infection. There were no signs of improper wound healing in the places where the implant was placed. Clinically, both in the skin of the operated ear and in the skin of the lower abdomen after hair removal, it was difficult to find the scar lines. Within the ear, the surgical sites could be palpated as a thickening on the skin, while the elasticity of the cartilages was maintained. There were no visible signs of surgical intervention in the form of scars within the skin of the lower abdomen, and the implantation site was recognized after comparing it with intraoperative pictures of the implantation site. By touch, the skin of the lower abdomen was smooth, maintaining its natural shape and consistency (Figure 2). There were no signs of inflammation in the form of redness, swelling, or exudate in the places of implantation (Figure 2).

### 3.2. Histopathological Evaluation

**Figure 3:**
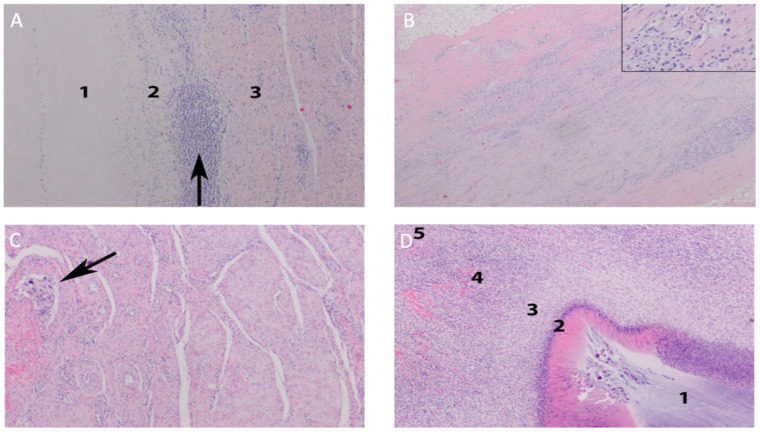
Auricle. Resorptive reaction to the implant. Zone 1: implant, zone 2: macrophagal infiltration, zone 3: fibrous scar tissue. Focal nodular cluster of lymphocytes (arrow) (**Panel A**). Auricle. Interstitial granules (insert) are visible between the thick bundles of fibrous scar tissue (**Panel B**). Body integument. Fibrous scar tissue formed by thick collagen-fibroblastic bundles. Focal resorptive granuloma (arrow) (**Panel C**). Body integument. Resorptive reaction to an implant. Zone 1: implant, zone 2: purulent necrosis, zone 3: macrophagal infiltration, zone 4: granulation tissue, zone 5: fibrous scar tissue (**Panel D**). Reproduced from [3].

Among the analyzed materials, in one case (animal no. 1) of the abdominal integuments, it was found that the implant was partially resorbed and suppurated on a microscopic level, demarcated by fibrosis. In the other two, the implants were completely resorbed and fibrous scar tissue was formed. Single focal granulomas were integrated into the surroundings of the scar and could have been reactions to sutures. In one ear implant (animal no. 3), the changes were similar to those of animal no. 1 in the abdominal tissues, i.e., a fragment of the implant surrounded by a histiocytic reaction was preserved, but without a granulocytic infiltration (no suppuration). In other cases, the effect of the process was the formation of scars, among which there was visible granulomatous inflammation of the interstitial type (Panels B–D in Figure 3).

In two of the analyzed cases, the presence of preserved cellulose was found (in one ear and in one case with the coatings). In one case (muscle, pig no. 2), the excised site did not contain any signs of BC or reaction to BC, and thus it was deemed as a “missed excision site”. Details of histopathological evaluation are presented in Table 1.

In pig no. 1, in the ear location, congestion was assessed as mild, chronic inflammation was assessed as medium, there was no purulent infiltration, fibrosis was of a medium degree, giant cells were present, and cellulose was absent.

In pig no. 2, in the ear location, congestion was assessed as mild, chronic inflammation was assessed as medium, there was no purulent infiltration, fibrosis was of a medium degree, giant cells were absent, and cellulose was also absent.

In pig no. 3, in the ear location, congestion was assessed as mild, chronic inflammation was assessed as high, there was no purulent infiltration, fibrosis was of a medium degree, and giant cells and cellulose were present.

In pig no. 1, in the muscle location, congestion was assessed as medium, chronic inflammation was assessed as high, purulent infiltration was present, fibrosis was of a medium degree, and giant cells and cellulose were present.

In pig no. 2, in the muscle location, the excision site was missed and the histopathological evaluation showed no signs of BC implantation.

In pig no. 3, in the muscle location, there was no congestion, chronic inflammation was assessed as slight, purulent infiltration was absent, fibrosis was of a medium degree, and giant cells and cellulose were absent.

## 4. Discussion

Modern surgery requires minimal damage to the body’s integuments in the treatment process. Reconstructive surgery using foreign materials aims to meet these requirements as much as possible. The literature describes many materials to fill in the resulting tissue defects. The basic method is to use the patient’s own tissues, but there are products such as AlloDerm, polyurethane, and polyethylene that are well researched and their use is well documented [11,12,13]. The usefulness of bacterial cellulose (BC) is very promising; however, its use is not yet well established and researched. The lack of the cellulase enzyme in animals means that it should not be broken down within their tissues. In humans, there is also no known mechanism for its degradation. This feature, as well as many others (e.g., the possibility to change the structure or shape), makes it possible to modify its application. Currently, the most advanced research is carried out on the use of BC in cardiac surgery to use it as heart valves [5] and in vascular surgery as substitutes for blood vessel walls [14]. Research assessing BC in reconstructive surgery is still scarce, which is partially bridged by the current work [15,16].

The method of producing bacterial cellulose is simple and relatively cheap, which makes it competitive with other implantable materials. The cellulose itself, as proved in previous studies, is indifferent to the host organism, does not cause rejection reactions, and should not be biodegradable. The bacterial cellulose used in the study was checked in terms of mechanical and physical conditions prior to implantation in the animal, as shown in previous studies [14].

There are a number of different methods for assessing implant biocompatibility in a donor found in the literature. It is possible to evaluate the graft by histological studies [14,17], biochemical studies [18], tissue and cell cultures [19], histochemical analyses [20], and perfusion studies of the whole organ [21]. Other physical features such as weight, elasticity, stiffness, mechanical malfunctions, elongation, and surface damage are possible to observe using electron microscopy [22]. Moreover, there are also newer technical possibilities utilizing radiology. X-ray [23], magnetic resonance imaging [24], and computed tomography [25] both with or without radiological contrast or radioactive isotopes have been helpful in observing possible inflammatory responses that could occur when using metallic implants and be caused by their decomposition and contamination of the operating site or even circulation in the bloodstream of a patient. Due to the high costs of most of the above-mentioned methods, the most commonly used method in experimental studies is a histological assessment with the use of hematoxylin–eosin dye.

In this study, clinical and histopathological examinations were used to evaluate the behavior of BC in living tissues. The assessed parts of pig ears and abdominal covers had an aesthetic appearance that did not differ from the normal ones, and the recipient sites did not show any signs of inflammation. The ear with the BC implant retained its stiffness and elasticity. Available cell-free matrices do not achieve the stiffness that can be obtained with BC, and therefore can be compared with artificial materials. In their case, however, there is a frequent risk of necrosis of living tissues in the area and the need to remove the implanted material. In the area of the ear, two out of three cases were resorbed with the formation of fibrous tissue, and in one case, there was no resorption. There were no signs of contamination with the features of rejection of the grafted material, which is often the case with typical artificial materials, such as polypropylene. These observations are confirmed in the work of Amorim et al., who studied the behavior of bacterial cellulose filling the defects of the nasal septum in rabbits. Although the bacterial strain used in their work was different, it also did not cause rejection, with a satisfactory degree of integration with the animal’s tissues^14^.

Research on the biocompatibility of implantable materials usually requires evaluation at different times, considering tissue responses to foreign bodies. The time of the progression of the changes was important for the assessment of the local inflammation and the appearance of the ear after the implantation of the cellulose body during the observation process. No published studies on the use of such material in the reconstruction of auricle cartilage have been found in the literature. Possible further studies on a larger group of animals with longer observation times should provide additional information about the breakdown of biocellulose observed in some samples and its influence on the shape of the ear. In the present study, it could only be noticed that the biocellulose shaped body was suitably plastic and could be easily handled. It is easy to place under the skin, providing a natural substitute and consistency compared to the rest of the cartilage parts of the ear. Thus, in the future, BC may be a satisfactory substitute for the missing fragments of cartilage parts of the auricle, and maybe even the septum or wings of the nose. Within the abdominal wall, there were no signs of surface defects, cavities resulting from the lack of tissues, or scar contractures. In the histopathological examination, one out of three implants placed within the rectus abdominis muscles was completely resorbed, producing fibrous tissue in this place, and another one was partially resorbed, also with the formation of fibrous tissue.

The performed histological examination showed a tendency for the BC implant to be incorporated into the scar tissue. It is properly accepted by the surrounding tissues of the animal without the excessive or pathological inflammatory response typical of a foreign body. The scar formed after implantation of the BC effectively joined the edges of the cavities and created a barrier separating healthy tissues from other artificial materials used in similar situations. We consider this moderate amount of scarred fibrous tissue to be beneficial, because mechanically, it is not a fully valuable but overall beneficial substitute for the tissue. This is also indicated as an advantage of BC material by other authors.

Important consideration arising from the present study is the biodegradability of BC, as this material, as mentioned, theoretically should not undergo biodegradation. However, as shown by the results of the present study, BC was found only in two of five excision sites upon histopathological examination. It is important to underline that the primary aim of BC use in reconstructive surgery is its clinical usefulness—specifically, the mechanical and aesthetic properties of the body site where BC was implanted. As shown in this study, these aims (proper filling of a defect) were properly achieved regardless of the result of histopathological examination. Therefore, while more research is needed to explain the course of BC degradation in mammals (as it can be expected that human subjects will show similar breakdown mechanisms as *Sus scorfa domesticus*), we can already draw conclusions that it is an adequate material for reconstructive properties.

There are also important considerations of this study. Primarily, this study included only three subjects with two sites each—six sites total. It could be desired to increase that number, but considering the ethics of animal studies, costs, and the consistent and promising results of the present study, the authors do not feel that it is needed to increase the number of subjects or to repeat the experiments. Further, this study did not explain the mechanism of BC degradation, but this process was observed. This warrants further studies to explain the specifics of this phenomenon. Finally, the main strengths of this study are the multidisciplinary team and the translational character of the study. As a group of various researchers— biomaterials experts, veterinarians, and plastic surgeons—we have made an important step toward the routine use of BC in plastic and reconstructive surgery.

## 5. Conclusions

The small number of in vivo trials performed does not allow far-reaching conclusions. The reaction to cellulose implants is probably modified by other coexisting factors, most likely infection. However, the correct formation of scar tissue in some animals by the resorptive granulomatous mechanism, leading to local scar formation, is a very desired result by the operator, especially in the event of possible connection and contact with other artificial materials often used in reconstructive surgery. The study showed that the implanted BC material is easy to process and easy to shape and model. Implanted into various tissues of the same animal, it meets the expectations of closing and filling the tissue defects created in it, even if BC itself is resorbed. Additional studies are warranted to further explore this topic in more complex clinical scenarios, such as possible interplays of BC with other implantable materials like silicone implants.

## Figures and Tables

**Table 1:** Histopathological evaluation of excised material.

	Congestion	Chronic Inflammation	Purulent Infiltration	Fibrosis	Presence of Giant Cells	Presence of Cellulose
Ear—Pig no. 1	1	2	0	2	1	0
Ear—Pig no. 2	1	2	0	2	0	0
Ear—Pig no. 3	1	3	0	2	1	1
Muscle—Pig no. 1	2	3	1	2	1	1
Muscle—Pig no. 2	Missed excision site					
Muscle—Pig no. 3	0	1	0	2	0	0

Congestion: 0—none, 1—slight, 2—medium, 3—high; chronic inflammation: 0—none, 1—slight, 2—medium, 3—high; purulent infiltration: 0—none, 1—present; giant cells: 0—absent, 1—present; fibrosis: 0—none, 1—low degree, 2—medium degree, 3—high degree; cellulose: 0—absent, 1—present.

## Data Availability

Data supporting reported results can only be found in this article.

## References

[1] Haddad A.G., Giatsidis G., Orgill D.P., Halvorson E.G. (2017). Skin Substitutes and Bioscaffolds: Temporary and Permanent Coverage. Clin. Plast. Surg..

[2] Ludwicka K., Kolodziejczyk M., Gendaszewska-Darmach E., Chrzanowski M., Jędrzejczak-Krzepkowska M., Rytczak P., Bielecki S. (2018). Stable composite of bacterial nanocellulose and perforated polypropylene mesh for biomedical applications. J. Biomed. Mater. Res. Part B Appl. Biomater..

[3] Jankau J., Błażyńska-Spychalska A., Kubiak K., Jędrzejczak-Krzepkowska M., Pankiewicz T., Ludwicka K., Dettlaff A., Pęksa R. (2022). Bacterial Cellulose Properties Fulfilling Requirements for a Biomaterial of Choice in Reconstructive Surgery and Wound Healing. Front. Bioeng. Biotechnol..

[4] Catanzano O., Quaglia F., Boateng J.S. (2021). Wound dressings as growth factor delivery platforms for chronic wound healing. Expert Opin. Drug Deliv..

[5] Kołaczkowska M., Siondalski P., Kowalik M.M., Pęksa R., Długa A., Zając W., Dederko P., Kołodziejska I., Malinowska-Pańczyk E., Sinkiewicz I. (2018). Assessment of the usefulness of bacterial cellulose produced by *Gluconacetobacter xylinus* E25 as a new biological implant. Mater. Sci. Eng. C Mater. Biol. Appl..

[6] Martínez Ávila H., Feldmann E.-M., Pleumeekers M.M., Nimeskern L., Kuo W., de Jong W.C., Schwarz S., Müller R., Hendriks J., Rotter N. (2015). Novel bilayer bacterial nanocellulose scaffold supports neocartilage formation in vitro and in vivo. Biomaterials.

[7] Jaroennonthasit W., Lam N.T., Sukyai P. (2021). Evaluation of carbon sources from sugar industry to bacterial nanocellulose produced by Komagataeibacter xylinus. Int. J. Biol. Macromol..

[8] Tapking C., Popp D., Branski L.K. (2019). Pig Model to Test Tissue-Engineered Skin. Methods Mol. Biol..

[9] Voipio H.-M., Baneux P., Gomez DeSegura I.A., Hau J., Wolfensohn S. (2008). Guidelines for the veterinary care of laboratory animals: Report of the FELASA/ECLAM/ESLAV Joint Working Group on Veterinary Care. Lab. Anim..

[10] Guillen J. (2012). FELASA guidelines and recommendations. J. Am. Assoc. Lab. Anim. Sci..

[11] Jansen L.A., De Caigny P., Guay N.A., Lineaweaver W.C., Shokrollahi K. (2013). The evidence base for the acellular dermal matrix AlloDerm: A systematic review. Ann. Plast. Surg..

[12] Jansen L.A., Macadam S.A. (2011). The Use of AlloDerm in Postmastectomy Alloplastic Breast Reconstruction: Part II. A Cost Analysis. Plast. Reconstr. Surg..

[13] Thoma D.S., Benić G.I., Zwahlen M., Hämmerle C.H.F., Jung R.E. (2009). A systematic review assessing soft tissue augmentation techniques. Clin. Oral Implant. Res..

[14] Amorim W.L., Costa H.O., De Souza F.C., De Castro M.G., Da Silva L. (2009). Experimental study of the tissue reaction caused by the presence of cellulose produced by Acetobacter xylinum in the nasal dorsum of rabbits. Braz. J. Otorhinolaryngol..

[15] Wippermann J., Schumann D., Klemm D., Kosmehl H., Salehi-Gelani S., Wahlers T. (2009). Preliminary Results of Small Arterial Substitute Performed with a New Cylindrical Biomaterial Composed of Bacterial Cellulose. Eur. J. Vasc. Endovasc. Surg..

[16] Ai F.-F., Mao M., Zhang Y., Kang J., Zhu L. (2019). Experimental study of a new original mesh developed for pelvic floor reconstructive surgery. Int. Urogynecology J..

[17] Högset O., Bredberg G. (2009). Plaster of Paris: Thermal Properties and Biocompatibility A Study on an Alternative Implant Material for Ear Surgery. Acta Oto-Laryngol..

[18] Sevastjanova N.A., Mansurova L.A., Dombrovska L.E., Slutskii L.I. (1987). Biochemical characterization of connective tissue reaction to synthetic polymer implants. Biomaterials.

[19] Vince D.G., Hunt J.A., Williams D.F. (1991). Quantitative assessment of the tissue response to implanted biomaterials. Biomaterials.

[20] Schadel A., Thun G., Stork L., Metzler R. (1993). Immunodiffusion and Immuno-histochemical Investigations on the Reactivity of Oxide Ceramic Middle-Ear Implants. ORL.

[21] Ribot E.J., Tournier C., Aid-Launais R., Koonjoo N., Oliveira H., Trotier A.J., Rey S., Wecker D., Letourneur D., Vilamitjana J.A. (2017). 3D anatomical and perfusion MRI for longitudinal evaluation of biomaterials for bone regeneration of femoral bone defect in rats. Sci. Rep..

[22] Pogorelova N., Rogachev E., Digel I., Chernigova S., Nardin D. (2020). Bacterial Cellulose Nanocomposites: Morphology and Mechanical Properties. Materials.

[23] Uo M., Watari F., Yokoyama A., Matsuno H., Kawasaki T. (2001). Tissue reaction around metal implants observed by X-ray scanning analytical microscopy. Biomaterials.

[24] Xu X., Gao J., Liu S., Chen L., Chen M., Yu X., Ma N., Zhang J., Chen X., Zhong L. (2021). Magnetic resonance imaging for non-invasive clinical evaluation of normal and regenerated cartilage. Regen. Biomater..

[25] Fernández M.P., Witte F., Tozzi G. (2019). Applications of X-ray computed tomography for the evaluation of biomaterial-mediated bone regeneration in critical-sized defects. J. Microsc..

